# LRMP: Layer Replication with Mixed Precision for spatial in-memory DNN accelerators

**DOI:** 10.3389/frai.2024.1268317

**Published:** 2024-10-04

**Authors:** Abinand Nallathambi, Christin David Bose, Wilfried Haensch, Anand Raghunathan

**Affiliations:** ^1^Elmore Family School of Electrical and Computer Engineering, Purdue University, West Lafayette, IN, United States; ^2^Argonne National Laboratory, Materials Science Division, IL, United States

**Keywords:** in-memory computing, analog accelerator, quantization, reinforcement learning, mixed integer linear programming

## Abstract

In-memory computing (IMC) with non-volatile memories (NVMs) has emerged as a promising approach to address the rapidly growing computational demands of Deep Neural Networks (DNNs). Mapping DNN layers spatially onto NVM-based IMC accelerators achieves high degrees of parallelism. However, two challenges that arise in this approach are the highly non-uniform distribution of layer processing times and high area requirements. We propose LRMP, a method to jointly apply layer replication and mixed precision quantization to improve the performance of DNNs when mapped to area-constrained IMC accelerators. LRMP uses a combination of reinforcement learning and mixed integer linear programming to search the replication-quantization design space using a model that is closely informed by the target hardware architecture. Across five DNN benchmarks, LRMP achieves 2.6–9.3× latency and 8–18× throughput improvement at minimal (<1%) degradation in accuracy.

## 1 Introduction

Deep Neural Networks (DNNs) have come to dominate the field of machine learning, and achieve state-of-the-art performance on a variety of complex tasks. However, the advancements in their capabilities have come at the cost of a steep growth in model size and computational complexity. Researchers have developed specialized digital accelerator architectures (Chen et al., 2016; Jouppi et al., 2018; Lie, 2022) and methodologies like quantization and pruning (Liang et al., 2021) that attempt to strike better tradeoffs between cost and functional performance. These implementations are, however, fundamentally limited by the memory bottleneck, as memory accesses are significantly more expensive than arithmetic operations.

In-Memory Computing (IMC) is a computing paradigm where the elementary operations of input vector-weight matrix multiplications in DNNs are performed within memory arrays, potentially alleviating the memory bottleneck. IMC systems have been designed and prototyped with various memory technologies, including SRAM (Zhang et al., 2017; Kang et al., 2020; Yin et al., 2020), DRAM (Gao et al., 2019), and emerging non-volatile memories (NVM) such as RRAM (Chi et al., 2016; Shafiee et al., 2016; Song et al., 2017), PCM (Burr et al., 2015; Khaddam-Aljameh et al., 2021; Narayanan et al., 2021) and STT-MRAM (Jain et al., 2018; Yan et al., 2018). In this work, we focus on IMC with emerging NVMs, where the weights are programmed into the NVM arrays as the conductance, of the memory device. To perform a vector-matrix multiplication (VMM) using such a memory array, the input vector can be presented simultaneously along multiple wordlines using digital-to-analog converters (DACs). The current that flows through each memory cell is the product of the conductance of the memory element (weight) and the wordline voltage (input). These currents naturally sum up at each bit line. These behaviors are dictated by Ohm's and Kirchoff's laws. These bit line currents can then be digitized using analog to digital converters (ADCs) to produce the input vector-weight matrix dot product.

Emerging non-volatile memories have a lot of desirable qualities that make them a good fit for in-memory computing. Their high density means that larger models can be stored, and their non-volatility eliminates the need for continuous power or refresh. However, their high programming costs coupled with their limited endurance make frequent weight re-programming undesirable. These factors make NVMs suitable for weight-stationary inference architectures where all the weights are programmed spatially across the chip and activations flow through and get processed by the appropriate arrays. A consequence of this approach is that the required area scales with the size of the network. While NVM arrays are compact, the peripherals required for IMC (ADCs and DACs) can be quite large, lowering the effective density (storage capacity per unit area) and thus, resulting in large area requirements. To mitigate the large area requirements, researchers have proposed bit-decomposed architectures (Shafiee et al., 2016; Ankit et al., 2019), in which weight bits are stored in spatially distinct arrays and input bits are processed serially, reducing the precision requirements of ADCs and DACs. As the area of the peripherals scale with their precision, the reduced precisions of ADCs and DACs in bit-decomposed architectures result in better effective density, thereby lowering area requirements of IMC accelerators.

While mitigating the area requirements is important, the system performance is also an important consideration. Despite the impressive peak performance offered by spatial IMC accelerators, the actual performance achieved can be significantly lower due to poor utilization caused by the non-uniformity in processing times across the layers of a DNN. Effective mapping techniques can help close the gap between peak and actual performance (Jain et al., 2023). *Layer replication*, which replicates bottleneck layers to facilitate tensor and data parallelism, can balance layer processing times and thus, improve performance. However, layer replication is not a trivial optimization. Finding the resources to replicate the layers, choosing the right layers to replicate, and the number of times to replicate the chosen layers, are all decisions that involve complex tradeoffs between area requirements and performance. Also, with growing model sizes, improving the performance of spatial architectures with layer replication becomes challenging since it exacerbates the area requirements.

Researchers have proposed various techniques to address the challenges of IMC accelerator design. For example, various mixed precision quantization techniques that assign specialized bitwidths to the weights and activations across the layers of a DNN using different optimization strategies (Huang et al., 2021; Kang et al., 2021; Meng et al., 2021b; Peng et al., 2022) have been shown to achieve significant compression of weight and activation footprints, resulting in area, speed and energy improvements. However, these techniques do not address the severe under-utlization of NVM tiles caused by the imbalance in processing times across the layers of DNNs. Others have proposed layer replication techniques to improve utilization that either do not address the question of where to find the resources to replicate layers (Rasch et al., 2019; Li et al., 2020; Li W. et al., 2023) or rely on design-time tradeoffs to accommodate the replicated layers (He et al., 2022). In contrast, we identify a novel synergy between **L**ayer **R**eplication and **M**ixed **P**recision quantization that can be exploited at compilation-time to improve the performance of DNNs on spatial IMC accelerators. We propose LRMP, an automated mapping framework that explores the quantization-replication design space to quantize layers selectively to free up resources and then replicate the right layers using the freed-up resources to improve performance. In summary, our contributions are as follows:

- We present LRMP, a novel framework that jointly performs mixed precision quantization and layer replication during mapping of DNNs to IMC accelerators.
- We propose a joint-optimization approach with (i) deep reinforcement learning based selection of precision for each layer in the network to maintain accuracy while conserving hardware resources, and (ii) linear programming based selective layer replication to redeploy the conserved resources in the IMC hardware to improve performance.
- We evaluate the LRMP framework on a benchmark suite of convolutional and fully-connected neural networks and achieve 2.6–9.3× latency improvement and 8–18× throughput improvement, at iso-area and near iso-accuracy.

The rest of the paper is organized as follows. We describe the process of mapping a neural network layer in an IMC system and discuss the implications of precision on resource requirements and latency in Section 2. We motivate the synergy between mixed precision and layer replication using an illustrated example in Section 3. We present the details of the LRMP framework in Section 4. We describe our experimental setup in Section 5 and present our results in Section 6. We discuss the contributions of our work in the context of existing related works in Section 7. Finally, we conclude the paper in Section 8.

## 2 Preliminaries

Vector-matrix multiplication (VMM) is an elementary operation in the evaluation of neural networks. In this section, we describe how a weight matrix can be mapped to multiple crossbar tiles and how these crossbar tiles can collectively perform a multiplication operation between an input vector and a weight matrix to produce an output vector. We also describe the latency and the number of crossbar tiles required for such an implementation of VMM.

Convolutional layers represent a common layer configuration used in DNNs. They are composed of a three-dimensional weight tensor array sliding across a three-dimensional input tensor, producing an output value for each patch of overlap. Convolutional layers are realized on IMC substrates by converting the weight tensor into a two-dimensional matrix and performing image-to-column lowering of the input tensor into a sequence of vectors. Then, the convolution output values can be produced by performing a sequence of VMMs.

Consider a convolution operation with *C* input features, *N* output features and a kernel size of *K*, producing output features of dimension *W* × *W*. The size of its lowered weight matrix is *K*^2^*C* × *N*. The input tensor is transformed into *W*^2^ vectors of length *K*^2^*C*. With a sequence of VMMs, *W*^2^ output vectors of length *N* are produced. It must be noted that the number of vectors can be quite high and it depends on the dimensions of the input, the filter kernel size *K*, the padding and the stride. For, example, in the first convolutional layer of the ResNet18 DNN, the input matrix has over 12,000 column vectors.

**Figure 1:**
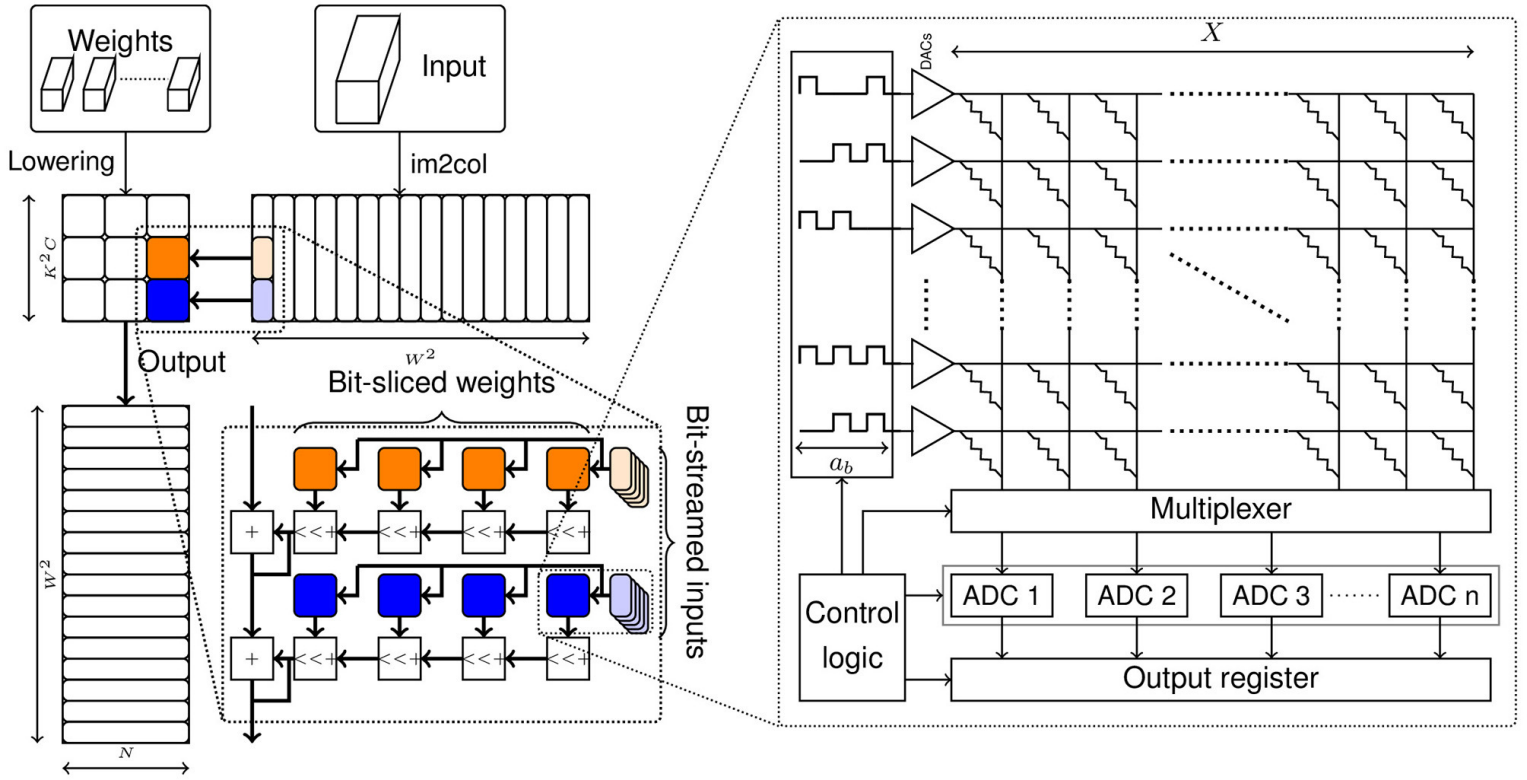
Realizing a VMM operation using crossbar arrays.

To map a convolutional layer to a crossbar, the weight tensor is first lowered to a two-dimensional matrix, as shown in Figure 1. The weight matrix is then segmented into multiple sub-matrices of size *X* × *X*, which denotes the size of the crossbar array or tile. To build a spatial architecture, each of these sub-matrices are mapped onto individual crossbar tiles. The number of tiles required to perform this spatial mapping is given by Equation 1.


(1) $$\begin{array}{l} \#\text{tiles}(K,C,N,X)=\left\lceil \frac{K^{2}C}{X}\right\rceil \times \left\lceil \frac{N}{X}\right\rceil \end{array}$$

As discussed in Section 1, crossbar arrays can be built with a variety of memory technologies. Also, these memory devices are designed to store a specific number of bits. The precision of the memory element is a matter of concern, as high precision elements have been shown to be more sensitive to process variations and conductance drift (Shim et al., 2021) and incur higher programming costs (Perez et al., 2021). Thus, low precision devices are desirable with regards to both accuracy and performance.

The achievable precision of the memory device needs to be reconciled with the required logical precision of the weights. If the device precision (*s*_*b*_) is less than the required weight precision (*w*_*b*_), the weight sub-matrices can be sliced into groups of *s*_*b*_ bits and then each slice can be mapped to a separate crossbar tile, as shown in Figure 1. It must be noted that the digital outputs corresponding to the bit-slices of the weight matrix need to be appropriately shifted and added to produce the final output. The number of tiles required considering a bit-sliced mapping is given by Equation 2.


(2) $$\begin{array}{l} \#\text{tiles}(K,C,N,X,w_{b},s_{b})=\left\lceil \frac{K^{2}C}{X}\right\rceil \times \left\lceil \frac{N}{X}\right\rceil \times \left\lceil \frac{w_{b}}{s_{b}}\right\rceil \end{array}$$

As discussed in Section 1, to perform a vector-matrix multiplication using crossbars, we must convert the input vector values to analog voltages using a digital-to-analog converter (DAC). The output currents of the crossbar array are then converted to digital using analog-to-digital converters (ADC). These peripheral circuits, especially ADCs, occupy a significant proportion of latency and, area and power budgets. The design choices of the number of ADCs per crossbar array, and ADC and DAC precisions are important considerations in the design of crossbar-based architectures. The number of ADCs can be chosen to be lesser than the number of columns and the ADCs can be time-multiplexed between multiple columns. In order to reduce the precision requirements of ADCs and DACs, we can stream the input vectors bit-by-bit and reduce the corresponding outputs with shift-add operations. The latency of performing VMMs required by a convolution layer in such a bit-streamed manner is given by Equation 3.


(3) $$\begin{array}{l} \text{lat}(W,a_{b},X,n_{ADC},t_{tile})=W^{2}\times t_{tile}\times \left\lceil \frac{X}{n_{ADC}}\right\rceil \times a_{b} \end{array}$$

where *n*_*ADC*_ is the number of ADCs per crossbar array, *t*_*tile*_ is the time elapsed between presenting an input to the tile and the ADCs producing their output, *a*_*b*_ is the number of bits required to represent the input vector values and *W*^2^ is the number of vectors.

Thus, both the hardware requirements and latency of a crossbar-based architecture depend on the precision of the weights and activations of the neural networks mapped onto them.

## 3 Motivation

In this section, we illustrate how mixed precision and layer replication greatly impact latency and throughput of DNN evaluation.

**Figure 2:**
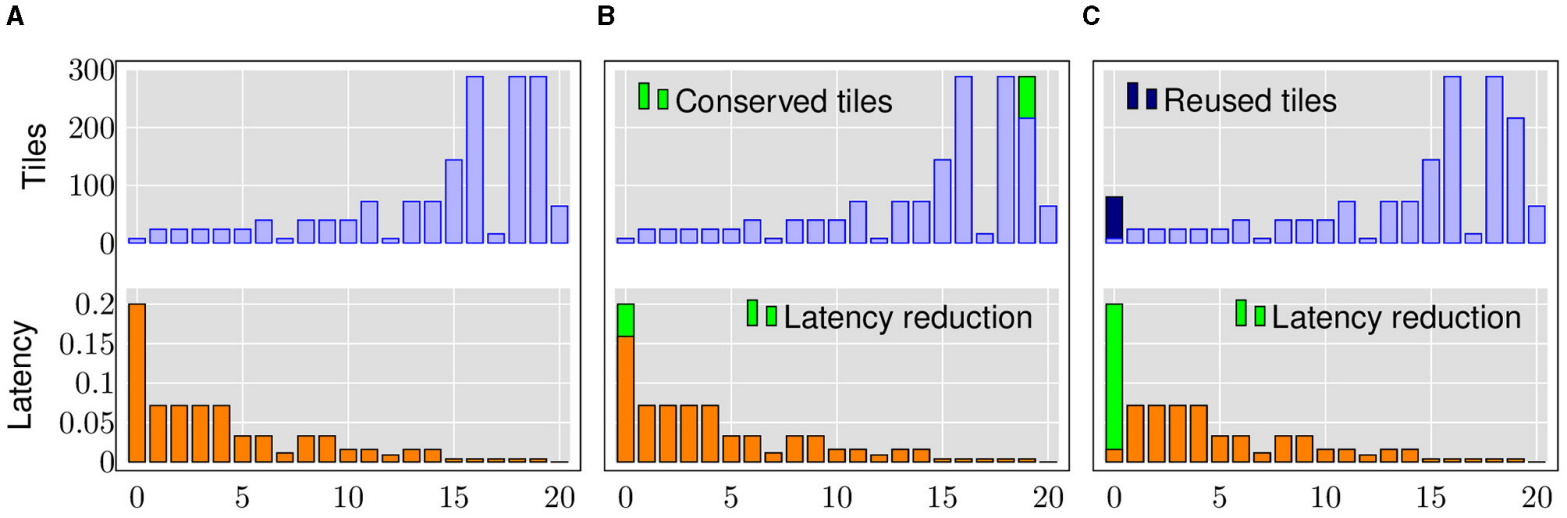
An experimental illustration of heterogeneous quantization and layer replication using ResNet18. **(A)** 8-bit baseline. **(B)** Selective Quantization. **(C)** Layer Replication.

Let us consider the baseline implementation of ResNet18 with 8-bit weights and 8-bit activations. As defined by Equation 2, the tile consumption of each layer in a spatial architecture depends on the size of the weight matrix, the logical weight precision (*w*_*b*_) and the physical memory device precision (*s*_*b*_). As shown in Figure 2A, we observe that different layers of the network have different latencies and tile requirements, as defined by Equations 3 and 2, respectively. In our evaluations, we use a device precision of 1-bit and a crossbar size of 256×256.

By selectively reducing the precision of weights in certain layers, we can take advantage of the bit-sliced implementation and reduce the number of tiles required by that layer. These conserved tiles can be used to replicate bottleneck layers in a neural network to process parts of the layer input in parallel, resulting in a latency reduction. Similarly, by selectively reducing the precision of input vectors, we can reduce the number of bits to be streamed to the crossbar arrays, resulting in proportional reduction of latency.

Let us consider reducing the weight precision of a resource-intensive layer and the input precision of the bottleneck layer to 6-bits. As shown in Figure 2B, we observe that 72 tiles are conserved. In addition, as explained by Equation 3, the latency of the bottleneck layer is reduced resulting in an overall 5.7% latency improvement and 1.33× throughput improvement.

If these newly freed-up tiles are used to naively replicate only the bottleneck layer, we can create 9 more copies of that layer. Thus, 10 input vectors of that layer can be processed in parallel, resulting in 25.5% improvement in total latency and 2.34× improvement in throughput, as illustrated in Figure 2C.

The above example illustrates that a trade-off exists between precision and latency in spatial IMC architectures. A few questions that arise related to this trade-off are:

### 3.1 How to choose the precision of each layer?

When choosing the precision of each layer, we need to consider its impact on the tile consumption, overall latency, and accuracy. The weight precision affects the bit-slicing factor, which is only one of the factors that determines the tile consumption of a layer (Equation 2). The other factors depend on the size of the weight matrix. Thus, it is important to choose the weight precision of each layer in a way that the number of tiles conserved is maximized. Similarly, the activation precision only affects the bit-streaming factor of Equation 3. The other factor is the number of input vectors to be processed. Thus, it is important to choose the activation precision of each layer in a way that the latency is minimized. Moreover, reducing the activation/weight precision of any layer in a neural network has implications for the overall accuracy of the network. Thus, it is important to choose the precision of each layer in a way that the overall accuracy is not compromised.

### 3.2 Where to repurpose the conserved tiles?

When choosing the replication factor of a layer, we need to consider its impact on the overall latency and tile consumption. The latency of a layer is a function of the number of input vectors and their precision, both of which can vary across the layers of a neural network. At the same time, the number of tiles required to replicate layers also varies based on the size of their weight matrices. Thus, it is important to choose the replication factor of each layer in a way that the utility of the conserved tiles is maximized, and the overall latency is minimized.

## 4 LRMP methodology

In this paper, we propose LRMP (Layer Replication through Mixed Precision), a framework that combines reinforcement learning (RL) and mixed integer linear programming (MILP) to jointly optimize latency/throughput and accuracy of DNNs realized on IMC hardware fabrics.

**Figure 3:**
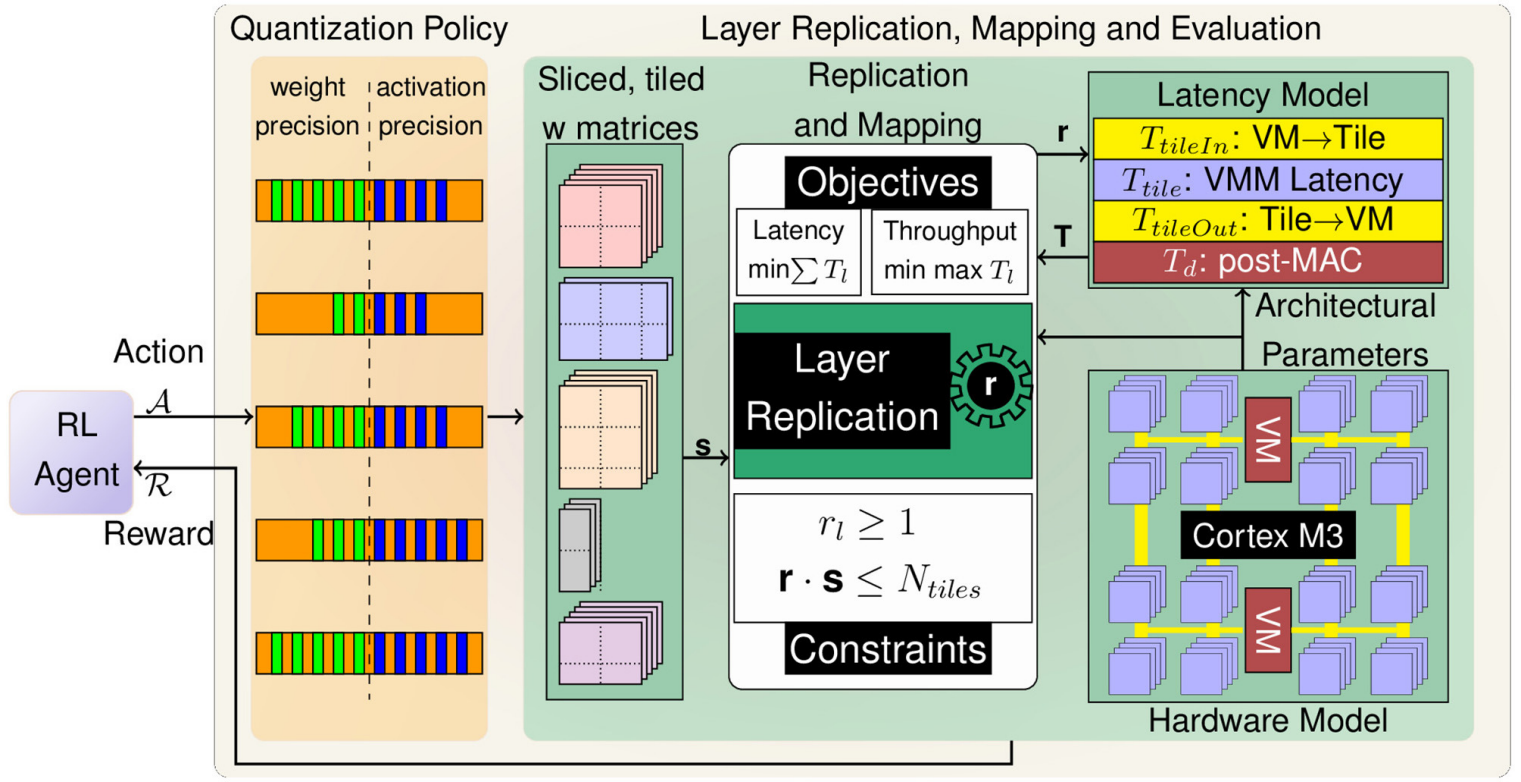
Overview of the proposed LRMP methodology.

As shown in Figure 3 LRMP is an iterative process with each iteration or episode consisting of two-steps: (1) an RL-agent choosing the precision of each layer in the DNN, and (2) an MILP-based optimizer choosing the replication factors of each layer. After each episode, the latency, throughput and accuracy of the network are evaluated and used to guide the RL-agent. In the remainder of this section, we describe the hardware modeling of an RRAM-based IMC accelerator, and then discuss how linear programming and reinforcement learning can be employed in tandem to quantize and replicate layers to jointly optimize accuracy and performance metrics under a chip capacity constraint.

### 4.1 Hardware model

LRMP is a joint optimization process that is designed to improve accuracy and latency/throughput achieved by DNNs on spatial in-memory accelerators. To estimate the performance metrics of evaluating DNNs on these spatial accelerators, we develop a simple and effective cost model that can be used to estimate latency and throughput of a DNN during the optimization process. The cost model is based on the compute-in-memory system developed by Chang et al. (2022), which consists of a Cortex M3 microprocessor, two vector modules for digital compute, and 288 crossbar tiles of dimension 256×256. Data transport is implemented using 8 lanes of 8-bit wide buses from the vector modules to the crossbar tiles and 8 lanes of 32-bit wide buses from the crossbar tiles to the vector modules. Each vector module has 8 lanes of parallel compute and 128KB of SRAM. The system is equipped with fine-grained power gating and each tile can be individually turned off. On account of the larger computer vision models used to benchmark the proposed approaches in this work, the cost model assumes a scaled-up version of this system with 5688 tiles and 40 vector modules, each with 64 lanes of parallel compute. Further details on the microarchitecture are provided in Section 5.

The latency of evaluating a DNN layer (*T*_*l*_) on this compute-in-memory system, described in Equation 4, comprises of 4 factors: *T*_*tileIn*.*l*_, which refers to the latency of transferring input vectors of layer *l* from vector modules to the respective tiles. This transfer is performed over eight 8-bit lanes, which are shared among 144 tiles. Similarly, *T*_*tileOut*.*l*_ represents the latency of transferring output vectors of layer *l* from crossbar tiles to the respective vector modules using eight 32-bit lanes shared by the same 144 tiles. The latency of performing Vector-Matrix-Multiplication (VMM) using crossbar tiles with temporally bit-streamed inputs and spatially bit-sliced weights of layer *l* is represented by *T*_*tile*.*l*_. Lastly, the post-VMM digital compute of layer *l* performed by vector modules has a latency of *T*_*d*.*l*_, which uses 64 lanes processing the output vectors of 144 tiles. It must be noted that each of these components is a function of the number of bits used to represent the input activations and weights of layer *l*.


(4) $$\begin{array}{l} T_{l}=T_{tileIn.l}+T_{tileOut.l}+T_{tile.l}+T_{d.l} \end{array}$$

With the latency of evaluating a layer defined, the latency of evaluating a DNN is the sum of the latencies of its constituent layers. The latency of evaluating a DNN with *L* layers is given by Equation 5.


(5) $$\begin{array}{l} T=\overset{L}{\underset{l=1}{\sum }}T_{l} \end{array}$$

The system is designed to operate with coarse-grained pipeline parallelism. Thus, the throughput of the system is defined by the maximum latency of any layer. The throughput of the system is given by Equation 6.


(6) $$\begin{array}{l} P=\frac{1}{\underset{l}{\max}\;T_{l}} \end{array}$$

The model described above is used in LRMP to perform analysis and exploration of the quantization and replication design space.

### 4.2 Optimizing layer replication using mixed integer linear programming

As discussed in Section 3, tiles can be freed up by selectively quantizing layers based on their tile footprint and selectively replicating layers based on their latencies. When a layer is replicated, the number of tiles and vector modules allocated to that layer is increased. Thus, for the said layer: (1) the total bandwidth available for data transfer is increased; (2) the amount of digital compute allocated is increased; and (3) the number of tiles available for performing the required VMM operations is increased. This results in a linear reduction in the latency of evaluating the layer.

If there are *r*_*l*_ instances of layer *l*, then the latency of evaluating the DNN is given by Equation 7.


(7) $$\begin{array}{l} T=\underset{l}{\sum }\frac{1}{r_{l}}\left(T_{tileIn.l}+T_{tileOut.l}+T_{tile.l}+T_{d.l}\right) \end{array}$$

Given a mixed precision quantization scheme, the total number of tiles required to have one instance of each layer (*s*_*l*_) is given by Equation 2. The total latency *T* can be optimized by carefully choosing the layer replication factors denoted by the vector **r**. The process of choosing the replication factors is naturally constrained by the total number of tiles available in the system (*N*_*tiles*_). This can be formulated as a constrained optimization problem, as shown in Formulation 8.


(8) $$\begin{array}{l} \underset{r}{\text{minimize}}\;\sum_{l}\frac{1}{r_{l}}(T_{tileIn.l}+T_{tileOut.l}+T_{tile.l}+T_{d.l})\\ \text{subject to}\\ r_{l}\geq 1,\\ \;\sum_{l}\left(r_{l}*s_{l}\right)\leq N_{tiles} \end{array}$$

The constraints ensure that there is at least one instance of each layer and the total number of allocated tiles doesn't exceed the number of tiles available (*N*_*tiles*_). Since *s*_*l*_ is constant for a given quantization scheme, the constraints are linear. However, the objective function is non-linear.

As defined by Equation 6, the throughput of the system is the inverse of the maximum latency across all layers. Thus, to maximize throughput, we need to minimize the maximum latency across all layers. Optimizing for throughput is, thus, a min-max problem. The optimization problem can therefore be re-written as shown in Formulation 9.


(9) $$\begin{array}{l} \underset{r}{\text{minimize}}\;M\\ \text{subject to}\\ \frac{1}{r_{l}}(T_{tileIn.l}+T_{tileOut.l}+T_{tile.l}+T_{d.l})\leq M\\ \text{subject to}\\ r_{l}\geq 1,\\ \sum_{l}\left(r_{l}*s_{l}\right)\leq N_{tiles} \end{array}$$

We introduce a dummy variable *M* and reformulate the optimization problem to minimize *M*, while ensuring that the latency of each layer does not exceed *M*. By constraining the latency of each layer to be no greater than *M* and minimizing *M*, we are effectively minimizing the maximum latency across all layers, which maximizes the throughput of the system.

These optimization problems are not automatically linear. However, we can employ linearization techniques (Asghari et al., 2022) to reformulate the optimization problem with linear constraints and objective function. Then, we solve the reformulated problem using an MILP solver.

### 4.3 Constraining the action space with performance budgets

The reinforcement learning framework used in this work is based on the work by Wang et al. (2019), which imposes a performance cost constraint on the action space of the RL agent. If the quantization policy prescribed by the RL agent does not meet the performance targets, it is modified by decreasing the bitwidths until the performance targets are met. While this approach is effective, it does not provide any insight into the tradeoffs between accuracy and performance. We restructure this approach to explore the tradeoffs between accuracy and performance by exponentially tightening the performance budget. This results in the RL agent exploring the space of quantization policies to not just meet a performance budget but also achieve better performance metrics.

### 4.4 Rewarding the RL agent with accuracy and performance metrics

In each episode of exploration, the RL agent is rewarded based on the quality of the quantization policy it prescribes. Wang et al. (2019) rewarded the RL agent based on the accuracy of the quantized DNN. In this work, we optimize the performance of the quantized DNN by using the layer replication technique. Thus, to achieve joint optimization, the RL agent is rewarded based on the accuracy and performance of the quantized DNN. The reward function is given by Equation 10.


(10) $$\begin{array}{l} R=\lambda \times \left(acc_{quant}-acc_{original}\right)+\alpha \times \left(1-T_{quant}/T_{original}\right) \end{array}$$

where, *acc*_*quant*_ is the accuracy of the quantized DNN, *acc*_*original*_ is the accuracy of the original DNN, *T*_*quant*_ is the latency of the quantized DNN and *T*_*original*_ is the latency of the original DNN when optimizing for latency. When optimizing for throughput, *T*_*quant*_ and *T*_*original*_ are latencies of the bottleneck layers of the respective DNNs. The hyperparameters λ and α control the relative importance of accuracy and performance in the reward function. The reward function is designed to encourage the RL agent to prescribe quantization policies that result in a quantized DNN that is optimized to balance accuracy and speed.

## 5 Experimental methodology

### 5.1 Microarchitectural details

As described in Section 4, this work is based on a scaled-up model of the compute-in-memory system fabricated by Chang et al. (2022). The microarchitectural parameters are listed in Table 1.

**Table 1:** Microarchitectural parameters.

**Parameter**	**Value**
eNVM	1T-1R RRAM
Tile size	256× 256
No. of tiles	5688
No. of vector modules	40
Device precision	1 bit
Row parallelism	9
DAC precision	1 bit
Column parallelism	8
ADC precision	4 bits
Avg. power per tile	130 μW
Clock frequency	192 MHz

The system is built using 1T-1R RRAM eNVM technology, with a tile size of 256x256 and a total of 5688 tiles. The system also includes 40 vector modules, each of which contains 64 lanes of parallel digital compute and 128 KB of SRAM. Each tile is equipped with eight 4-bit Flash ADCs and 256 1-bit DACs. To prevent partial sum quantization and mitigate other non-idealities, only 9 rows are activated at a time. The system is clocked at 192 MHz.

The energy consumption of the system is modeled with three components: power consumed by the RRAM tiles, the energy cost of reading and writing the activations to the on-chip SRAM buffers, and the power leaked by the SRAMs. Each RRAM tile is reported to consume an average power of 130 μW (Chang et al., 2022). The SRAM blocks are modeled using CACTI.

While we evaluate LRMP on a specific architecture, the proposed techniques are not limited to it. The proposed optimizations are applicable to any bit-decomposed IMC architecture (Shafiee et al., 2016; Ankit et al., 2019; Zhu et al., 2019), and are otherwise agnostic to the underlying hardware.

### 5.2 Methods

#### 5.2.1 Reinforcement learning

As described in Section 4, the reinforcement learning framework used in this work is based on the hardware-aware quantization tool proposed by Wang et al. (2019). The method consists of two phases: exploration and finetuning. In the exploration phase, the agent explores the action space to find a good policy based on the performance budget and rewards provided, as described in Section 4. The trajectory of the exploration phase is discussed in Section 6.3.

After the exploration phase, the DNN is quantized with the mixed precision scheme found by the agent. In the finetuning phase, the DNN is trained with the quantized weights and activations to recover any accuracy lost to quantization.

#### 5.2.2 Mixed integer linear programming

Given a quantization policy prescribed by the RL agent, the mixed integer linear programming step is used to find the replication factors that optimize the performance of the system. Optimization objectives of both latency (*latencyOptim*) and throughput (*throughputOptim*) are implemented. The baseline for each network in the benchmark suite is the implementation with 8-bit weights and activations. Thus, the layer replication is performed with a constraint that the total number of tiles used is no more than the baseline. This is a design choice to ensure that performance is optimized without increasing area. An ablation study has been performed and described in Section 6.5 to show the effectiveness of our LRMP method with and without this design constraint.

### 5.3 Benchmarks

The proposed LRMP approach has been evaluated on a set of DNN benchmarks trained on the ImageNet and MNIST datasets. The baseline of comparison for each benchmark is the implementation with 8-bit weights and activations. The benchmarks are listed in Table 2, along with the number of tiles required by the baseline implementation. The multilayer perceptron (MLP) is trained on the MNIST dataset, with 4 hidden layers of 1024, 4096, 4096 and 1024 neurons respectively. The ResNets are finetuned on the ImageNet dataset with pre-trained weights. While we limit our evaluation to DNNs trained for classification tasks, we believe the proposed techniques are broadly applicable to any quantized DNN (Dettmers et al., 2022; Li X. et al., 2023). Also, the proposed techniques do not place any limits on the number of bits used for quantization.

**Table 2:** DNN benchmarks.

**Benchmark**	**Dataset**	***N*_*tiles*_**
MLP	MNIST	3,232
ResNet18	ImageNet	1608
ResNet34	ImageNet	2968
ResNet50	ImageNet	3376
ResNet101	ImageNet	5688

It must be noted that, besides quantization, analog non-idealities such as noise, conductance drift, device-to-device variation etc. have not been modeled in this work. However, modeling these non-idealities (Jain et al., 2020; Lu et al., 2021; Roy et al., 2021) and developing compensation techniques (Charan et al., 2020; Meng et al., 2021a; Jain and Raghunathan, 2019) are areas of active and ongoing research and we believe these effects are not an impediment to the principal contributions of this work.

## 6 Results

In the sub-sections of this section, we first present the latency, throughput and energy improvements achieved by LRMP. We then present results that provide insights into the RL-based exploration process. We also show a layer-wise breakdown of how latencies are optimized and an ablation study that analyses the sensitivity of the layer replication methodology to area constraints.

### 6.1 Latency and throughput improvements

**Figure 4:**
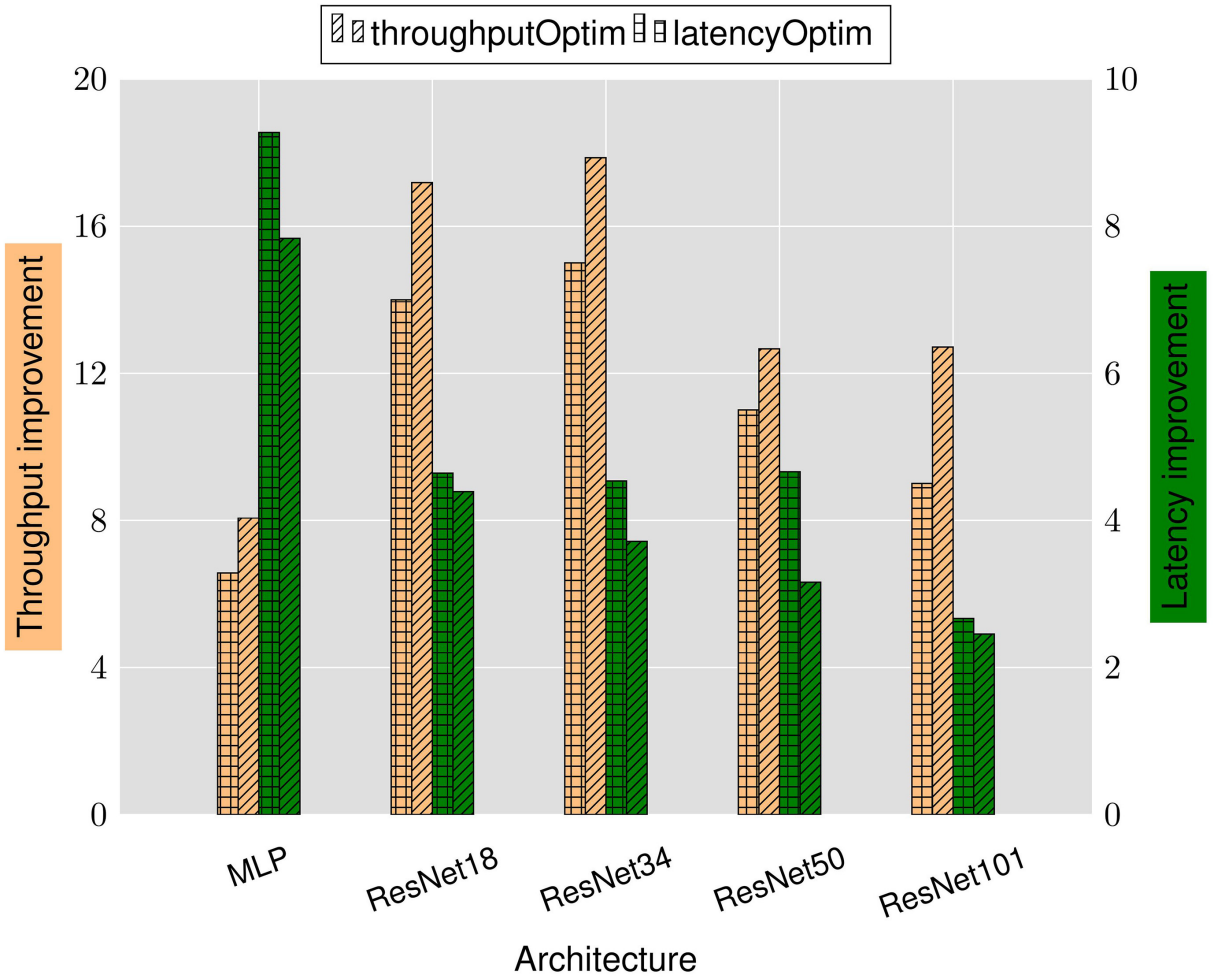
Latency and throughput improvements achieved by LRMP.

Figure 4 reports the latency and throughput improvements achieved by the LRMP framework. As explained in Section 5, the improvements are reported with respect to fixed-precision baseline networks with 8-bit weights and activations.

We observe 2.6–9.3× reduction in latency and 6.6–15× improvement in throughput while optimizing for latency (denoted as *latencyOptim*) across the suite of benchmark DNNs. Similarly, we observe 8–18× improvement in throughput and 2.5–7.8× reduction in latency while optimizing for throughput (denoted as *throughputOptim*). These improvements are obtained with accuracy loss of less than 1% after finetuning with the quantization policies determined by LRMP.

### 6.2 Energy improvements

**Figure 5:**
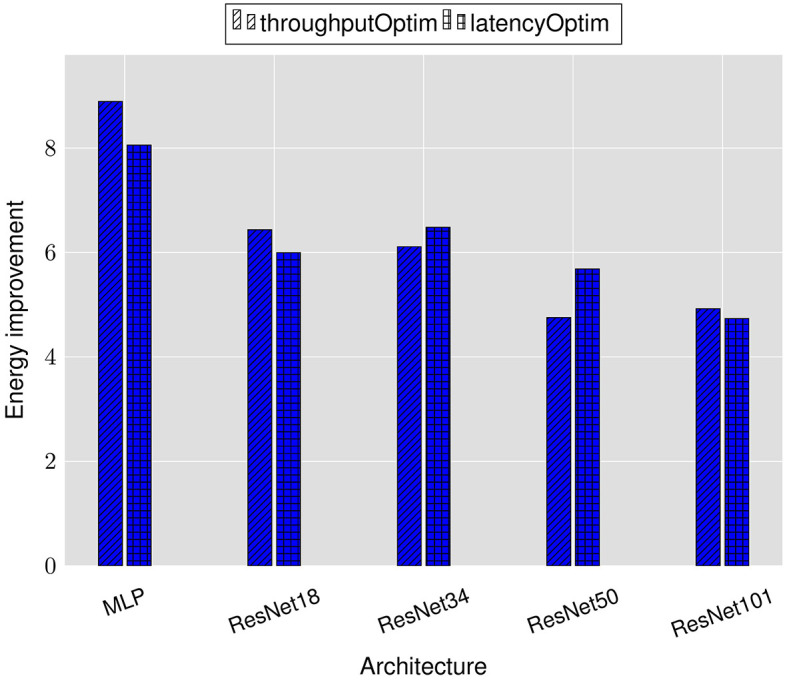
Energy improvements achieved by LRMP.

Although LRMP explicitly optimizes for throughput or latency, it achieves energy improvements as a result of more efficient DNN execution on the IMC substrate. Figure 5 shows the energy improvements achieved by LRMP. We observe 4.75–8.9× improvement in energy consumption while optimizing for throughput and 4.7–8× energy improvement while optimizing for latency.

### 6.3 Studying joint optimization of accuracy and performance

**Figure 6:**
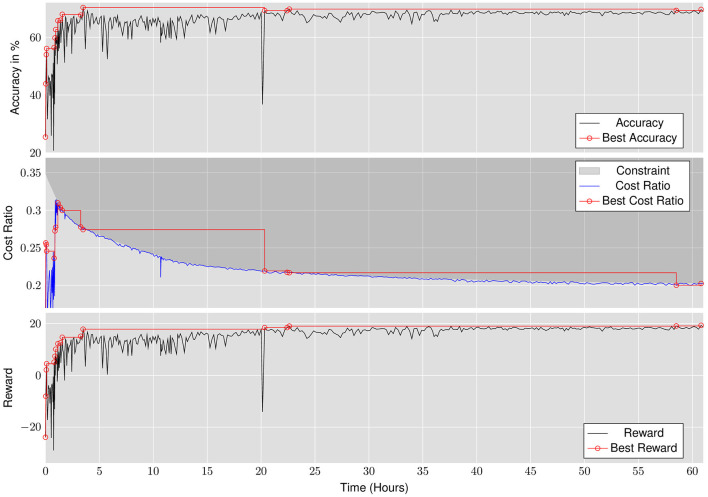
Trajectory of RL agent jointly optimizing ResNet18 for accuracy and latency.

As discussed in Section 4, the proposed approach jointly optimizes for accuracy and performance by rewarding the RL agent with an affine combination of accuracy and performance metrics, and by continuously tightening the constraints placed on the action space. Figure 6 shows the trajectory of the RL agent performing latency optimization for ResNet18. The exploration is started with a lenient performance budget of 0.35× baseline latency and exponentially tightened to 0.2× baseline latency. Over the course of the exploration, the agent finds quantization policies that achieve upto 5× improvement in latency with layer replication while also improving the accuracy.

### 6.4 Layer-wise breakdown

**Figure 7:**
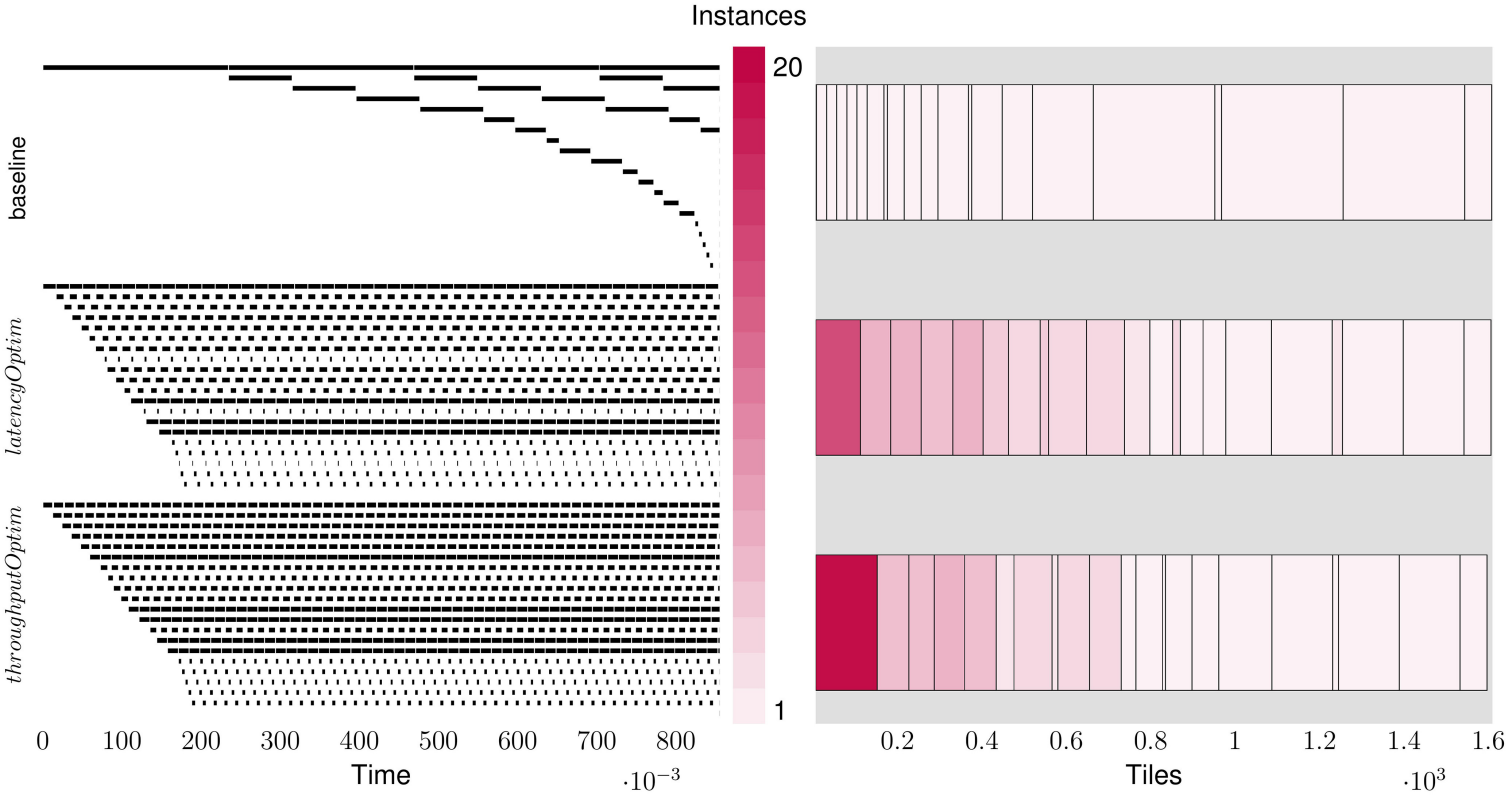
Layer-wise breakdown of latencies and tiles for ResNet18 for the baseline and while optimizing for latency (*latencyOptim*) and throughput (*throughputOptim*).

**Figure 8:**
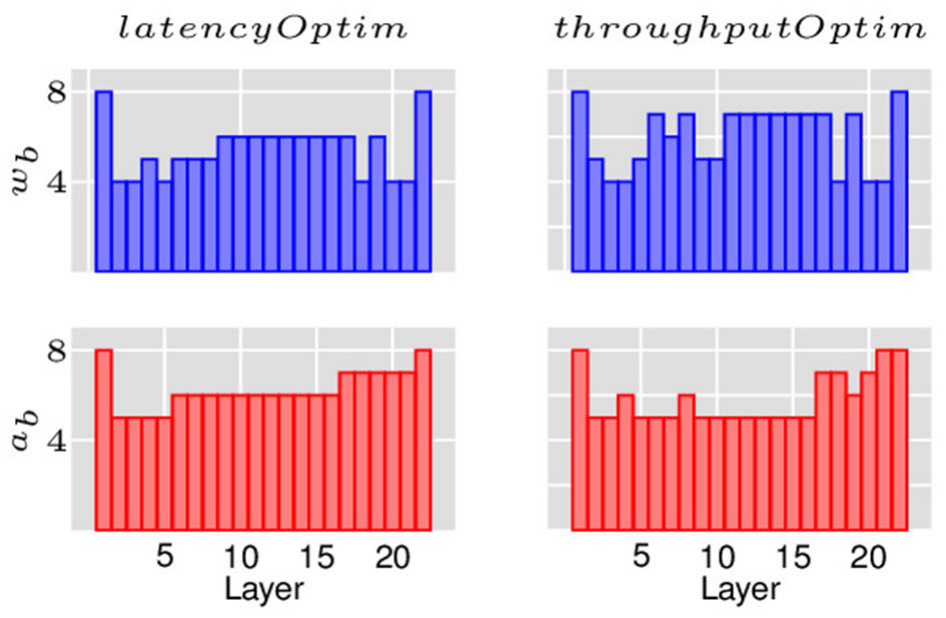
Quantization policies found by LRMP for ResNet18 while optimizing for latency and throughput.

As discussed in Section 4, the layer replication can be performed by optimizing for either latency or throughput. The two objectives have different implications on the latencies and tile consumptions of each layer and thus, latency and throughput outcomes for the overall network. Figure 7 shows the layer-wise breakdown of latencies and tiles for ResNet18 for the baseline implementation as well as the LRMP implementation while optimizing for latency and throughput. Figure 8 shows the quantization policies found by LRMP for ResNet18 while optimizing for latency and throughput.

In the baseline case, we observe that the latency of the network is bottlenecked by the first layer, which happens to consume very few tiles. When the layers are replicated for latency optimization (*latencyOptim*), the total latency is reduced by a factor of 4.6×, while the latency of the bottleneck layer is reduced by 14× as 13 more copies of that layer are created. In the throughput optimization mode (*throughputOptim*), the total latency is reduced by a slightly smaller factor of 4.4×, while the latency of the bottleneck layer is reduced by a larger factor of 19× as 18 more copies of that layer are created. This is understandable, because the bottleneck layer is solely responsible for determining throughput, while all layers contribute to latency. It can be observed that LRMP significantly improves tile utlization by balancing the pipeline stages through quantization and replication, resulting in an energy efficiency of 820 GOPS/s/W with *throughputOptim*, improving from 127 GOPS/s/W.

### 6.5 Analysis of sensitivity to chip area

As discussed in Section 5, the layer replication methodology is performed with an area constraint based on the fixed precision baseline i.e., *N*_*tiles*_ in the optimization constraints is equal to the number of tiles required by the fixed-precision 8-bit baseline network *baseline*_*tiles*. We note that a different design choice, based on the chip area and power budgets, could result in the relaxation or tightening of this tiles constraint.

**Figure 9:**
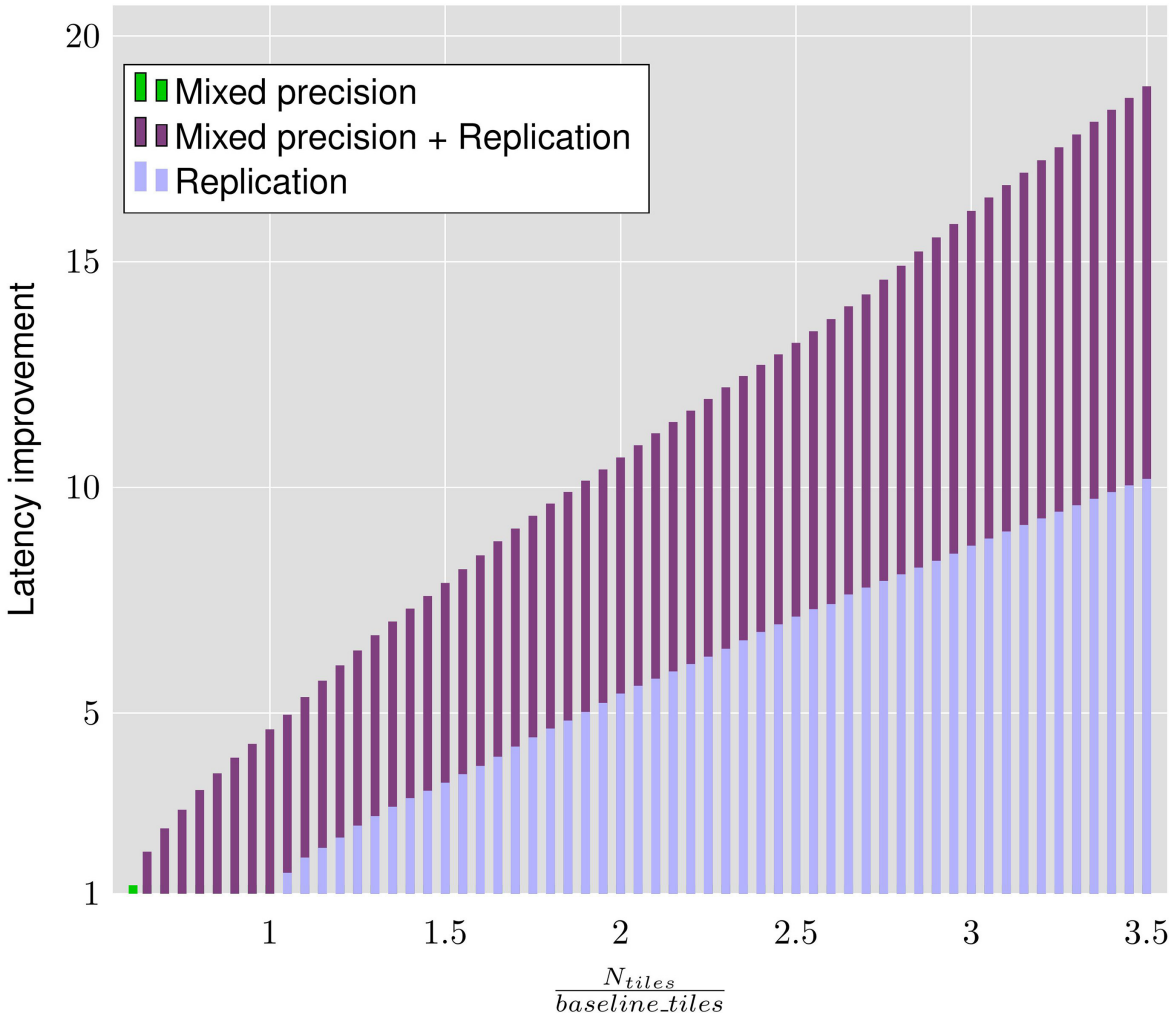
Latency improvements achieved by the proposed approach on ResNet18 with different area constraints.

Figure 9 shows the sensitivity of the latency improvements achieved by LRMP to different area constraints for the ResNet18 DNN. We perform this analysis by setting *N*_*tiles*_ to different ratios of *baseline*_*tiles* and using LRMP to perform only quantization, only replication, and joint quantization and replication. In other words, we study the behavior of LRMP by tightening the tiles constraint below the number of tiles required by the baseline or by relaxing the tiles constraint by making more tiles available in the system, while also using only one of the two optimization dimensions of LRMP.

Because of the model compression naturally achieved by mixed precision, with *only* mixed precision, we achieve 15.75% reduction in latency while using 39% fewer tiles than the baseline. When we employ mixed precision *and* layer replication, we observe latency reductions of 48% while using 35% fewer tiles than the baseline.

We note that layer replication can be performed even without mixed precision, if more tiles are available. We employ *only* layer replication with the baseline ResNet18 and observe 32% reduction in latency while using 5% more tiles than the baseline. It should be noted that when the tiles constraint is tightened, latency reductions are not possible without mixed precision, as there are not enough tiles for even a single copy of all the layers. Also, when all the tiles in the system are used, using mixed precision *and* layer replication achieves 46% lower latency compared to using only replication.

## 7 Related works

In this section, we discuss related previous work in the areas of quantization and pruning for DNN implementation on IMC hardware, as well as optimized mapping of DNNs to IMC substrates.

### 7.1 Quantization

Quantization is the process of reducing the number of bits used to represent numbers, which naturally adds distortions in the form of quantization noise. Quantizing weights and activations of neural networks is a common technique to reduce the model size and improve performance and energy efficiency. Pruning is a technique that removes connections and neurons in a neural network to improve sparsity. These complementary techniques have been widely explored to optimize neural network implementations. Quantization and pruning techniques have also been applied specifically to the context of in-memory computing. Peng et al. (2022) proposed a neural architecture search-based approach to perform mixed precision quantization in a crossbar-aware manner. Kang et al. (2021) proposed a methodology to perform energy-aware quantization using a genetic algorithm. Huang et al. (2021) proposed a methodology that performs quantization at the tile granularity powered by reinforcement learning. Meng et al. (2021b) proposed a quantization and pruning framework for efficient RRAM IMC implementations.

### 7.2 Mapping optimization

Mapping neural networks to IMC architectures is a complex problem. Li et al. (2020) proposed an approach to optimize the mapping of multimodal neural networks to IMC hardware to improve throughput. Gopalakrishnan et al. (2020) developed a methodology to design convolutional neural networks that would map better to crossbar architectures. Peng et al. (2019) proposed a weight mapping methodology that would improve data reuse of convolutional layers on crossbars. He et al. (2022) proposed a methodology to replicate layers in a neural network based on area freed-up by optimization of peripheral circuitry.

### 7.3 Optimization of peripheral circuitry

The peripheral circuits of crossbar tiles i.e., the DAC and ADC systems are crucial parts of IMC designs. ADCs contribute to a large portion of the power and area budgets, and are thus, a major bottleneck in the design of IMC systems. Various optimized IMC designs have been proposed that address these bottlenecks. Jiang et al. (2021) discusses an ADC design that implements shifts and adds in the analog domain. Saxena et al. (2022) proposed replacing ADCs with 1-bit sense amplifiers and training neural networks to be tolerant to such aggressive partial sum quantization. He et al. (2022) proposed an approach of decreasing the row parallelism to reduce the area overhead of ADCs and thus, improve the effective density of IMC chips.

To the best of our knowledge, LRMP is the first work that proposes a synergistic methodology that combine the benefits of mixed precision quantization and mapping optimization to jointly optimize the performance and accuracy of IMC-based neural network accelerators. LRMP is also the first work that proposes a linear programming-based approach to perform layer replication in IMC systems. Furthermore, circuit optimizations of tile peripheral are largely complementary to LRMP, and can be used to further improve the performance.

## 8 Conclusion

In-memory computing is a promising technology for accelerating neural networks by performing vector matrix multiplications within memory arrays. We propose LRMP, a method to synergistically perform layer replication and mixed precision quantization to improve performance of DNNs when mapped to area-constrained IMC accelerators. Our experiments suggest that LRMP can achieve considerable improvements in latency, throughput and energy consumption with similar accuracy compared to 8-bit fixed point implementations.

## Data availability statement

Publicly available datasets were analyzed in this study. This data can be found here: https://www.image-net.org/, http://yann.lecun.com/exdb/mnist/.

## Author contributions

AN: Conceptualization, Methodology, Software, Validation, Visualization, Writing – original draft, Writing – review & editing. CB: Conceptualization, Writing – review & editing. WH: Methodology, Validation, Writing – review & editing. AR: Conceptualization, Funding acquisition, Methodology, Project administration, Resources, Supervision, Validation, Writing – original draft, Writing – review & editing.
